# Specificity in plant-mycorrhizal fungal relationships: prevalence, parameterization, and prospects

**DOI:** 10.3389/fpls.2023.1260286

**Published:** 2023-10-20

**Authors:** Tyler W. d’Entremont, Stephanie N. Kivlin

**Affiliations:** Department of Ecology and Evolutionary Biology, University of Tennessee, Knoxville, TN, United States

**Keywords:** specialist, generalist, mutualism, selectivity, mycorrhizas

## Abstract

Species interactions exhibit varying degrees of specialization, ranging from generalist to specialist interactions. For many interactions (*e.g.*, plant-microbiome) we lack standardized metrics of specialization, hindering our ability to apply comparative frameworks of specificity across niche axes and organismal groups. Here, we discuss the concept of plant host specificity of arbuscular mycorrhizal (AM) fungi and ectomycorrhizal (EM) fungi, including the predominant theories for their interactions: Passenger, Driver, and Habitat Hypotheses. We focus on five major areas of interest in advancing the field of plant-mycorrhizal fungal host specificity: phylogenetic specificity, host physiology specificity, functional specificity, habitat specificity, and mycorrhizal fungal-mediated plant rarity. Considering the need to elucidate foundational concepts of specificity in this globally important symbiosis, we propose standardized metrics and comparative studies to enhance our understanding. We also emphasize the importance of analyzing global mycorrhizal data holistically to draw meaningful conclusions and suggest a shift toward single-species analyses to unravel the complexities underlying these associations.

## Introduction

In natural environments, species vary in the number and fidelity of biotic interactions, ranging from many interactions with low fidelity (“generalists”), to few interactions with high fidelity (“specialists”) (Bebber and Chaloner, 2022). The flexibility of these terms has resulted in their wide implementation and acceptance, but precise measures underlying these groupings are not often used. This has hindered researcher’s ability to apply comparative frameworks of specificity across niche axes or organismal groups. For example, defining a threshold of specificity to classify an organism as a niche specialist and across how many niches axes this specificity must occur are still open questions in mycorrhizal community ecology (*e.g.*, Davison et al., 2021). Determining components of species specificity is paramount as species face increasing extinction and extirpation pressure from climate change, habitat fragmentation, and species invasion (Lenoir et al., 2008), and identifying and mitigating constraints to organismal acclimation and adaptation to global change is key to preserving current biodiversity (Gábor et al., 2022).

Nowhere are the concerns of specialization leading to extirpation under global change more troubling than in communities that remain undersampled, microorganisms. Arbuscular mycorrhizal (AM) fungi and ectomycorrhizal (EM) fungi are the two most common microbial functional groups on Earth, forming symbiotic interactions with over 90% of plant species (Smith and Read, 2008). Both groups of fungi acquire and distribute limiting nutrients to plants in exchange for plant photosynthate (Smith and Read, 2008). The coupling of mycorrhizal fungi and plants plays a key role in shaping both communities and their global distributions (Neuenkamp et al., 2018). Despite both AM and EM fungi being root symbionts of plants, there are significant differences in morphology, function, and plant hosts with which they associate (Fei et al., 2022). Perhaps the most well-known difference between these two groups of fungi is the physiological structures associated with nutrient transfer between plants and themselves. Arbuscular mycorrhizal fungi penetrate plant cells and form characteristic structures call arbuscles, where they transfer resources between the plant host and fungus (Smith and Read, 2008). In EM fungi, nutrient exchange occurs from an extracellular Hartig net around the host plant root tips, without the need for an endosymbiotic structure (Smith and Read, 2008). The acquisition of nutrients from these two groups also differ, with AM fungi “scavenging” inorganic nutrients in soils and EM fungi “mining” organic nutrients (Fei et al., 2022). In terms of the host plants for these groups, the guild of AM fungi broadly associations with a diversity of plant species spanning many functional groups whereas the guild of EM fungi mostly associate with trees and a few shrub species, mainly in temperate and boreal forests (Smith and Read, 2008). Differences in dominant mycorrhizal fungal associations can affect biogeochemical cycles (Averill et al., 2019), soil carbon storage (Averill et al., 2014), and plant biodiversity (Fei et al., 2022) across ecosystems.

AM fungi represent an important group that have been well studied due to their effects on plant performance and diversity through mutualisms (Koziol and Bever, 2017; Begum et al., 2019) and their global distribution in terrestrial ecosystems (Kivlin et al., 2011; Davison et al., 2015; Davison et al., 2022; Vasar et al., 2022). Several theories have arisen to explain the distribution of mycorrhizal fungi, and while these theories mostly focus on AM fungi, they may apply to EM fungi even given the numerous differences in life strategies between these guilds. The Driver Hypothesis proposes that AM fungi are a key driver in maintaining and explaining plant diversity by selective association with plants and by doing so they shape the aboveground plant communities (Hart et al., 2001). Some support comes from studies showing plants with stronger associations with AM fungi have higher survival rates and are more competitive in nutrient-poor soils (Johnson et al., 2003; d’Entremont et al., 2021a), while others have shown that AM fungi can increase plant diversity by increasing the total number of species that can coexist in a given area (Van Der Heijden and Scheublin, 2007). In contrast, the Passenger Hypothesis proposes that although AM fungi are important for plant growth and survival, they do not play a significant role in regulating plant diversity or community composition and that plant community composition has control over this interaction (Grman, 2012). At the community level, mixtures of plants species can develop unique AM fungal communities based on the flora present, suggesting that plants may be involved in regulating mycorrhizal composition (Zobel and Öpik, 2014; Neuenkamp et al., 2018). Alternative to the former theories, the Habitat Hypothesis iterates neither the plants nor AM fungi are driving community structure, but instead environmental gradients control the covariation of these communities. At coarse resolution, studies have shown that climatic zones influence AM fungal community composition (Kivlin et al., 2011) and that pH can be a useful predictor of variation in AM fungal communities (Lekberg et al., 2011). Given these large differences in the three hypotheses on how plant-mycorrhizal fungal symbioses are shaped, we may observe vastly different patterns host specificity. If AM fungi or plants drive the partnership, we would expect much higher levels of host specificity than if abiotic factors control covariation. While we understand the basic principles of each of these mechanisms, there is still a stark need for elucidation of the relative importance each of these mechanisms play in mycorrhizal fungal response to changing environmental conditions.

For our perspective, we focus on five areas we believe are important for future research to better understand plant host specificity of AM and EM fungi. Although these groups of organisms are distantly related, we may be able to apply similar frameworks to understand their interactions with plant hosts. Here, we present an overview of our current understanding of 1) phylogenetic specificity; 2) plant host physiological specificity; 3) functional specificity; 4) habitat specificity; and their consequences for 5) mycorrhizal fungal-mediated plant rarity. We also provide standardized metrics to proceed with comparative studies of this nature (**Box 1**). Understanding whether these aspects of plant-fungal specialization are present will inform us how these interactions may shift under changing climates.

**Box 1:**
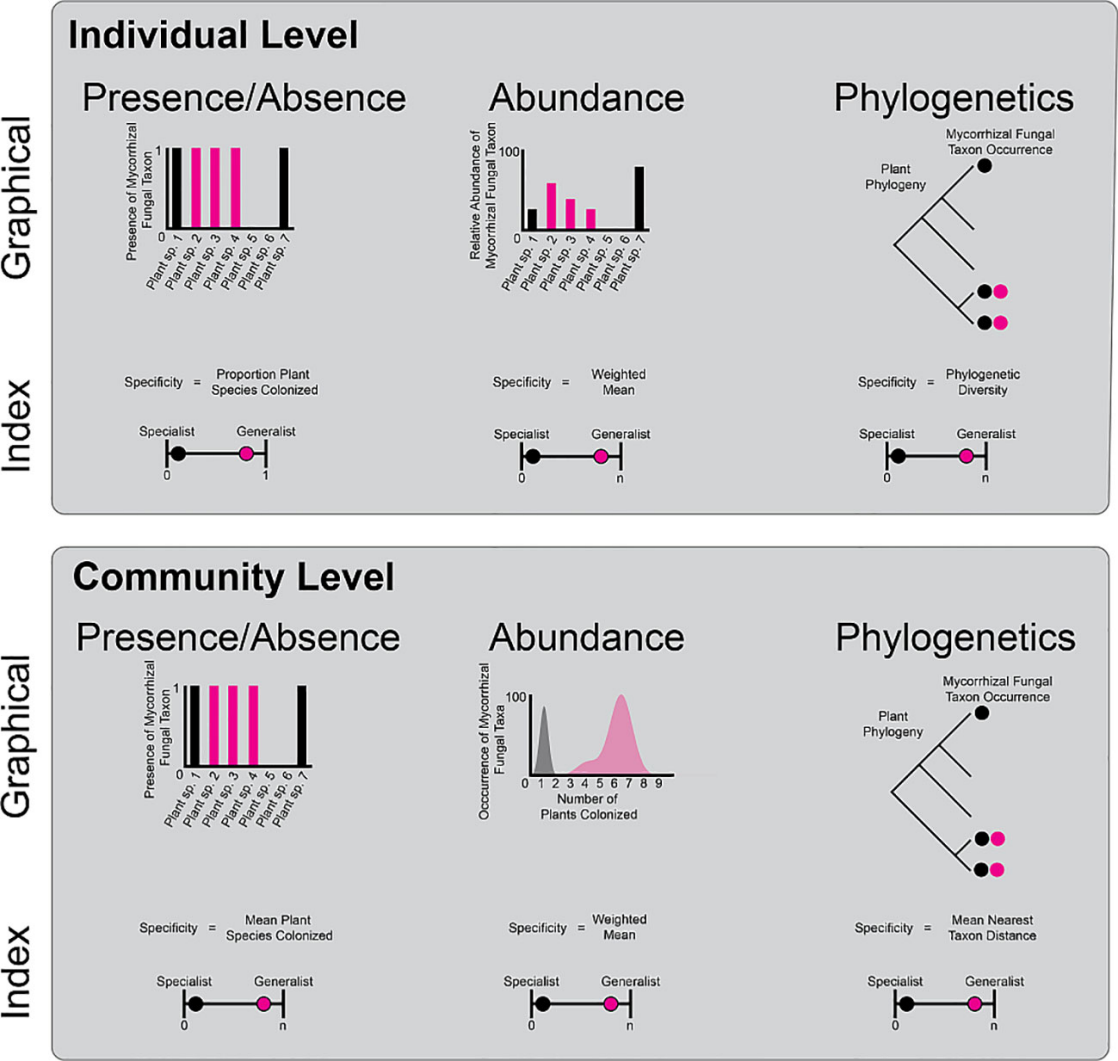
Specificity can be calculated in many ways and at multiple levels of biological organization. Two of the main ways that organisms can specialize are at the individual and community level. Individual level At the individual level, mycorrhizal fungal taxa may occur on one, few, or many plant taxa. The specialization of mycorrhizal fungi on plant species could be calculated at the proportion of plant species colonized in a given location or region. This presence/absence-based specialization index varies between near zero (specialist) and one (generalist). However mycorrhizal species can colonize plant roots at different rates. Thus, accounting for the differential abundance of colonization among plants (*e.g.*, as read numbers or biomass) adds an extra level of realism. To understand how different the abundance of colonization is among plant hosts, one could calculate the weighted mean colonization, with large, weighted means indicating generalism and low weighted means indicating high specificity of colonization rates on one to few plant species. Mycorrhizal fungi may colonize closely related plant species or colonize plant species across the plant phylogeny. Specialist mycorrhizal fungi may colonize plant species within one clade and therefore have low phylogenetic diversity (high specificity), whereas generalist mycorrhizal fungi may colonize plants across the phylogeny and have high phylogenetic diversity (low specificity) Community level These metrics (presence/absence, abundance, and phylogenetic diversity) can be applied to mycorrhizal fungal communities as well. For presence/absence metrics, a generalist mycorrhizal fungal community would have a large number of mean plant species colonized and a specialist mycorrhizal fungal community would have a small number of mean plant species colonized. Similarly, when examining abundance, generalist mycorrhizal fungal communities would have a high weighted mean number of plant species colonized, whereas a specialist mycorrhizal fungal community would have a low weighted mean number of species colonized. Finally, multiple metrics can be used to calculate the average phylogenetic distance among members of a mycorrhizal fungal community across the phylogeny. In this example, mean nearest taxon distance (MNTD; Webb, 2000, the distance between the two closest plant species both colonized by the same fungus, can categorize generalist mycorrhizal fungal communities (large MNTD) and specialist mycorrhizal fungal communities (small MNTD). There are many other ways to examine community level specialization including network metrics of bipartite plant and mycorrhizal fungal networks (Fodor, 2013), multiple alternative phylogenetic statistics, such as mean pairwise distance (MPD) (Webb, 2000), and dispersion of community composition in multivariate space (Anderson et al., 2006). Given the number of studies of plant-mycorrhizal fungal associations across habitats, it is timely to begin comparing specificity using multiple indices at both the mycorrhizal fungal individual and community levels. These comparative studies will create a lens into which habitats, plant traits, plant evolutionary histories, and plant functional types promote specialization of mycorrhizal fungi, improving forecasts of vulnerable habitats and taxa for conservation efforts under global change.

## Phylogenetic specificity

**Figure 1:**
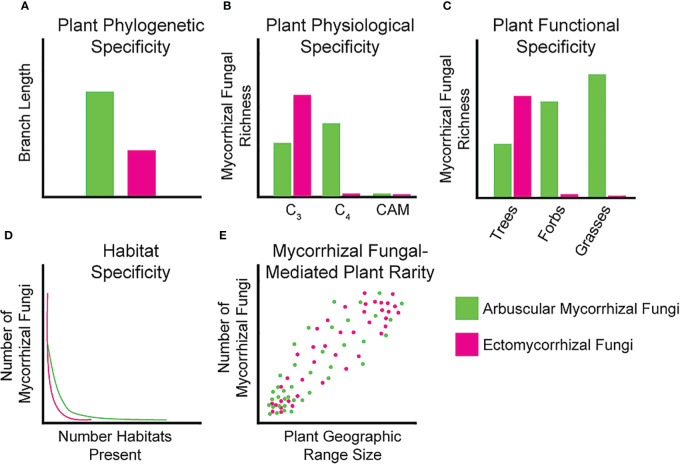
Idealized graphs showing our current knowledge of specificity between mycorrhizal fungi and host plants. **(A)** Phylogenetic diversity can be measured as plant branch length colonized by a given mycorrhizal fungal taxa, where AM fungi are proposed to colonize a larger breadth of the plant phylogeny compared to EM fungi; **(B)** Specificity of mycorrhizal fungi partitioned by host photosynthetic physiology. AM fungal richness should be higher on C_4_ plants whereas EM fungal richness should be higher on C_3_ plants; **(C)** Mycorrhizal fungal taxa richness of plant functional groups, AM fungal richness should be higher on all functional groups except trees; **(D)** Number of mycorrhizal fungi found in different habitats. AM fungi should colonize more habitats than EM fungi given the larger host breadth of AM fungi; **(E)** Number of mycorrhizal fungi correlated to plant geographic range indicating fewer mycorrhizal fungi may restrict geographic range. This effect should be consistent between AM and EM fungi.

Phylogenetic host specificity is the tendency for mycorrhizal fungal taxa to associate with closely related plant host species. We can view this as specificity in plant branch length along a phylogeny (**Figure 1A**) or within specific plant hierarchical levels (*e.g.*, family, genus, or species) to determine if any patterns exist that could play a role in shaping host specificity. Phylogenetic specificity could imply mycorrhizal fungal partners only associate with specific plant lineages, which may reflect the historical interactions of these fungi and their plant hosts either due to host specificity or vicariance and dispersal limitation of plants and mycorrhizal fungi (Dong et al., 2021). For AM fungal communities, there is currently mixed support for host selectivity at the plant species level with some studies suggesting generalism (Öpik et al., 2010; Davison et al., 2011; Davison et al., 2015), and others suggesting more specialist interactions (Kivlin et al., 2021; Kivlin et al., 2022; Ramana et al., 2023). There is, however, some evidence of a positive correlation between plant phylogenetic distance and the taxonomic dissimilarity of AM fungal assemblages (Dong et al., 2021). Plant root AM fungal communities can differ among different plant families in the same habitat/site, adding some support to the possibility of phylogenetic specificity (Torrecillas et al., 2012). For EM fungi, more evidence for phylogenetic specificity has been reported. For example, EM fungal *Suillus* species almost exclusively associate with taxa from the plant family *Pinaceae* (Lofgren et al., 2018). Additionally, a considerable number of EM fungal taxa have strong indications of host specificity at both the family and genus levels and plant phylogeny is often one of the best predictors of EM community composition at regional scales (Molina et al., 1992; Miyamoto et al., 2022). Studying the presence of host-symbiont interactions in locations diverse in taxa may shed some light on whether specific mycorrhizal fungal taxa associate with a diversity of plants or whether specific associations exist for some species. Accompanying data from greenhouse inoculation trials within a taxonomic level or between taxonomic levels can strengthen the findings of specificity from fieldwork. Finally, studies must isolate the same mycorrhizal fungal taxa from the roots of the same plant species across habitats to disentangle plant-driven versus habitat-driven specificity. Together, these data would provide a strong foundation to tease apart these complex interactions.

## Plant host physiological specificity

For plant physiology we focus on the photosynthetic pathway used by the plant host species of mycorrhizal fungi (**Figure 1B**). Host physiology may affect their ability to associate with mycorrhizal fungal partners that may be adapted to certain plant traits such photosynthetic pathway (Davison et al., 2020). Plant photosynthetic pathways differ in the amount and timing of carbon fixation and delivery to belowground mutualists. C_3_ plants fix continuously but may not be able to fix as much carbon under warm and dry conditions, whereas C_4_ plants can provide more consistent carbon supplies under these environmental stressors (Edwards and Walker, 1983). CAM plants, while rarely sampled, should also provide more consistent carbon under warm and dry conditions but only fix carbon at night (Töpfer et al., 2020). Fungal assemblages of AM fungi in C_4_ plants tend to have higher richness in taxa and C_4_ plants associate with fairly closely related AM taxa (Hoeksema et al., 2010; Davison et al., 2020). It is worth noting that beta diversity is higher for C_3_ plants at higher taxonomic levels, indicating that although the number of AM species they associate with is lower, AM fungi are more variable among C_3_ plant hosts (Davison et al., 2020). To our knowledge, AM fungi are rarely investigated in CAM plants, possibly owing to the fact that AM fungal biomass decreases in desert ecosystems in favor of dark septate endophyte symbioses (Rudgers et al., 2022). Similarly, investigating physiological specificity in EM fungi has not been conducted, likely due to the rarity of EM-associating C_4_ tree species (only existing in one genus *Euphorbia*) (Young et al., 2020).

## Functional specificity

Specificity of mycorrhizal fungi for different functional groups is another area of great interest, especially for AM fungi. We could expect to see specificity in this symbiosis in terms of which plant functional groups the AM fungi are able to associate with (*e.g.*, trees, shrubs, forbs, or grasses) due to differences in root morphology, rhizodeposits or other plant functional group-specific environmental conditioning (Davison et al., 2020). For EM fungi, which associate almost exclusively with trees, this form of specificity may be difficult to investigate (**Figure 1C**). Given the lack of functional diversity in observed plant host associates of EM fungi, little is known about how they may associate with other functional groups under different environmental conditions. Root traits of different functional groups can significantly affect mycorrhizal fungal assemblages due to different nutrient acquisition and conservation strategies, growth rates, and morphology (Sweeney et al., 2021). Composition of AM fungal assemblages differ among grasses, forbs, and trees with AM fungal richness being highest in grasses compared to the other groups (Sepp et al., 2019; Davison et al., 2020). Accentuating this, approximately 70% of virtual taxa (VTX) are unique to one plant functional group and only 4% of VTX are shared by all three functional groups (Yang et al., 2012). Isolating these taxa that exhibit the ability to transcend functional groups and inoculating a variety of diverse plants can provide understanding on which traits allow these taxa to overcome obstacles in these associations.

## Habitat specificity

Strong evidence exists to show that habitat filtering is a likely mechanism for mycorrhizal fungal species distributions (Kivlin et al., 2011; Davison et al., 2022; Vasar et al., 2022). Some plant species have different AM fungal communities in different habitats or site conditions, which may provide evidence that habitat filtering and abiotic factors play a big role in AM fungal community composition and persistence at sites (Li et al., 2010; d’Entremont et al., 2021b). Using tropical montane gradients, AM fungal assemblages have been related to the heterogeneity of habitats (soil textures) and not by the plant communities (Vieira et al., 2019). Ectomycorrhizal community composition is also significantly affected by edaphic properties of different habitat types, with narrower and more specialized habitats having the highest levels of EM specialization (**Figure 1D**) (Gerz et al., 2018; Arraiano-Castilho et al., 2021). Current research has identified key areas for high priority conservation efforts, specifically the tropical regions for AM and EM fungi (Tedersoo et al., 2022). Growing hosts and symbionts in differing soil conditions within controlled environments may elucidate mechanisms of environmental filtering and how it affects the survival of symbionts, adding valuable information to supplement existing research.

## Mycorrhizal fungal-mediated plant rarity

Although few studies correlate the effect of mycorrhizal symbiont range on the range of their plant host communities, these often obligate interactions may limit suitable plant habitat, enforcing a mechanism of plant rarity (Klironomos et al., 2011). Several mechanisms have been proposed for how plant rarity occurs with the prominent ones being distribution limitations, restrictions of range and usable habitats, and scarcity of symbionts (Phillips et al., 2011). Since rare plants often occur in habitats that are threatened or unique, they may utilize mycorrhizal fungal taxa specialized for these habitats as described in the previous section (**Figure 1E**) (Bothe et al., 2010). Additionally, most studies investigating rare plants and mycorrhizal fungi have relied heavily on root colonization and less on identifying the fungal symbionts (Bothe et al., 2010). Increasing focus on mycorrhizal fungal interactions with rare plant taxa may be especially important for elucidating cases of strong plant host specificity and when trying to stabilize these plant populations under current climate change regimes.

## Implications

Our current approaches have yet to capture the complexity of plant-mycorrhizal fungal interactions with too few studies sampling the same plants at multiple locations, which has hindered our ability to decouple the effect of plant host and habitat (Kokkoris et al., 2020). Without a detailed understanding of how these plant-mycorrhizal fungal distributions are controlled (Driver, Passenger, or Habitat), host specificity in mycorrhizal associations will continue to be a challenging question to answer. Additionally, while large data repositories (*e.g.*, MaarjAM, Opik et al., 2010) now exist, utilizing these data repositories can be difficult due to inconsistencies in reporting and differences in measured factors. Based on our current knowledge, cohesive and standardized experimental approaches are needed to allow for better comparisons between future studies to answer questions relating to host specificity. We propose a set of standardized specificity metrics in **Box 1**.

## Conclusion

Given the prominent literature on plant-mycorrhizal interactions and the “mixed-bag” of results that have been produced, global mycorrhizal data needs to be analyzed wholistically to determine whether we can draw any conclusions at a broad scale. Analyzing single species instead of fungal assemblages, as has been done in the majority of large-scale studies, may be one promising way forward to understand the magnitude of plant host specificity versus habitat specificity at global scales.

## Data availability statement

The original contributions presented in the study are included in the article/supplementary material. Further inquiries can be directed to the corresponding author.

## Author contributions

TWD: Conceptualization, Investigation, Methodology, Validation, Visualization, Writing – original draft, Writing – review & editing. SNK: Conceptualization, Investigation, Methodology, Project administration, Resources, Supervision, Visualization, Writing – original draft, Writing – review & editing.
